# Treatment Access for Gastrointestinal Stromal Tumor in Predominantly Low- and Middle-Income Countries

**DOI:** 10.1001/jamanetworkopen.2024.4898

**Published:** 2024-04-03

**Authors:** Edward Lloyd Briercheck, J. Michael Wrigglesworth, Ines Garcia-Gonzalez, Catherina Scheepers, Mei Ching Ong, Viji Venkatesh, Philip Stevenson, Alicia A. Annamalay, David G. Coffey, Aparna B. Anderson, Pat Garcia-Gonzalez, Michael J. Wagner

**Affiliations:** 1 Division of Hematology, University of Washington, Seattle; 2 now with Dana-Farber Cancer Institute, Boston, Massachusetts; 3 The Max Foundation, Seattle, Washington; 4 The Max Foundation, Buenos Aires, Argentina; 5 The Max Foundation, Johannesburg, South Africa; 6 The Max Foundation, Selangor, Malaysia; 7 The Max Foundation, Mumbai, India; 8 Division of Clinical Biostatistics, Fred Hutchinson Cancer Center, Seattle, Washington; 9 Division of Hematology, University of Miami, Miami, Florida; 10 Bill & Melinda Gates Medical Research Institute, Cambridge, Massachusetts; 11 Division of Oncology, University of Washington, Seattle; 12 Clinical Research Division, Fred Hutchinson Cancer Center, Seattle, Washington

## Abstract

**Importance:**

Gastrointestinal stromal tumor (GIST) is a rare cancer treated with the tyrosine kinase inhibitors imatinib mesylate or sunitinib malate. In general, in low- and middle-income countries (LMICs), access to these treatments is limited.

**Objective:**

To describe the demographic characteristics, treatment duration, and survival of patients with GIST in LMICs treated with imatinib and sunitinib through The Max Foundation programs.

**Design, Setting, and Participants:**

This retrospective database cohort analysis included patients in 2 access programs administered by The Max Foundation: the Glivec International Patient Assistance Program (GIPAP), from January 1, 2001, to December 31, 2016, and the Max Access Solutions (MAS) program, January 1, 2017, to October 12, 2020. Sixty-six countries in which The Max Foundation facilitates access to imatinib and sunitinib were included. Participants consisted of patients with approved indications for imatinib, including adjuvant therapy in high-risk GIST by pathologic evaluation of resected tumor or biopsy-proven unresectable or metastatic GIST. All patients were reported to have tumors positive for CD117(c-kit) by treating physicians. A total of 9866 patients received treatment for metastatic and/or unresectable disease; 2100 received adjuvant imatinib; 49 received imatinib from another source and were only included in the sunitinib analysis; and 53 received both imatinib and sunitinib through The Max Foundation programs. Data were analyzed from October 13, 2020, to January 30, 2024.

**Main Outcomes and Measures:**

Demographic and clinical information was reported by treating physicians. Kaplan-Meier analysis was used to estimate time to treatment discontinuation (TTD) and overall survival (OS). An imputation-based informed censoring model estimated events for patients lost to follow-up after treatment with adjuvant imatinib. Patients who were lost to follow-up with metastatic or unresectable disease were presumed deceased.

**Results:**

A total of 12 015 unique patients were included in the analysis (6890 male [57.6%]; median age, 54 [range, 0-100] years). Of these, 2100 patients were treated with imatinib in the adjuvant setting (median age, 54 [range 8-88] years) and 9866 were treated with imatinib for metastatic or unresectable disease (median age, 55 [range, 0-100] years). Male patients comprised 5867 of 9866 patients (59.5%) with metastatic or unresectable disease and 1023 of 2100 patients (48.7%) receiving adjuvant therapy. The median OS with imatinib for unresectable or metastatic disease was 5.8 (95% CI, 5.6-6.1) years, and the median TTD was 4.2 (95% CI, 4.1-4.4) years. The median OS with sunitinib for patients with metastatic or unresectable GIST was 2.0 (95% CI, 1.5-2.5) years; the median TTD was 1.5 (95% CI, 1.0-2.1) years. The 10-year OS rate in the adjuvant setting was 73.8% (95% CI, 67.2%-81.1%).

**Conclusions and Relevance:**

In this cohort study of patients with GIST who were predominantly from LMICs and received orally administered therapy through the GIPAP or MAS programs, outcomes were similar to those observed in high-resource countries. These findings underscore the feasibility and relevance of administering oral anticancer therapy to a molecularly defined population in LMICs, addressing a critical gap in cancer care.

## Key Points

**Question:**

Can oral anticancer therapy be distributed to patients with acceptable clinical outcomes through a global access program for the treatment of gastrointestinal stromal tumor (GIST)?

**Findings:**

In this cohort study of 12 015 patients, the median overall survival (OS) with imatinib treatment for patients with unresectable or metastatic GIST was 5.8 years, and the median time to treatment discontinuation (TTD) was 4.2 years. The median OS with sunitinib as second-line therapy for patients with metastatic or unresectable GIST was 2 years, the median TTD was 1.5 years, and the imputed 10-year OS rate for patients receiving imatinib in the adjuvant setting was 74%.

**Meaning:**

These findings suggest that patients with GIST who were predominantly from low- and middle-income countries and received orally administered therapy through a large global access program had clinical outcomes similar to those observed in patients treated in high-resource countries.

## Teaser

This cohort study analyzes the demographic characteristics, treatment duration, and survival of patients who received imatinib and/or sunitinib for gastrointestinal stromal tumors through global access programs.

## Introduction

Gastrointestinal stromal tumors (GIST) are mesenchymal tumors of the gastrointestinal tract.^1,2,3^ Imatinib, a tyrosine kinase inhibitor (TKI) targeting c-kit and platelet‐derived growth factor receptor α kinase activity, revolutionized GIST treatment but has been studied almost exclusively in high-income countries.^4,5,6,7,8,9,10,11,12,13,14^ Compared with a historical median overall survival (OS) of less than 8 months before the availability of imatinib mesylate,^15^ the median OS is now over 4 years in the metastatic setting.^4,5,6,7^ Other TKIs have since attained approval by regulatory bodies for the treatment of metastatic GIST after progression during or intolerance to imatinib treatment. The first was sunitinib malate, which was approved based on improved progression-free survival (PFS) in a placebo-controlled phase 3 study.^16,17^ A modeling of OS in this study estimated a 33-week improved OS with sunitinib compared with placebo.^16,17^ Retrospective studies of clinical datasets have shown that outcomes for patients treated with imatinib and subsequently with other TKI therapies are similar to outcomes seen in randomized clinical trials.^18,19^ After the success of imatinib in the unresectable or metastatic setting, additional studies demonstrated efficacy in the adjuvant setting.^8^ Based on these data, imatinib is the standard treatment for unresectable or metastatic GIST and for patients with resected high-risk GIST. Sunitinib is the standard second-line therapy after progression during or intolerance to imatinib treatment.

Despite these advances, implementation of GIST therapy in low- and middle-income countries (LMICs) has been limited. The resources and barriers to treatment within individual health care systems in LMICs are highly variable. Limitations include the high cost of treatment and the need for adequate laboratory infrastructure for both initial diagnosis and treatment monitoring.^20^ One solution is to increase access to cancer medicines through centralized structured programs such as those administered by The Max Foundation.

Between 2001 and 2017, The Max Foundation partnered with Novartis AG to administer the Glivec International Patient Assistance Program (GIPAP). The Max Foundation was instrumental in developing the systems necessary to receive and assess physician requests on behalf of individual patients and track the time on treatment of each patient over time, allowing the organization to keep track of the status of thousands of patient cases, their treating physicians, and the volume of drug supply needed.^21^ In 2017 the organization launched Max Access Solutions (MAS) to drive the evolution of the single-drug program into a multidrug and multicompany approach. Through MAS, The Max Foundation is able to make multiple medications that are approved by the US Food and Drug Administration or the European Medicines Agency, but not locally registered, available to physicians at cancer-treating institutions for patients who have been diagnosed with an approved indication of the product and have no other way to access the necessary medication. The programs by Novartis AG and The Max Foundation were initially focused on access to imatinib for the treatment of chronic myeloid leukemia (CML).^22^ On recognition of imatinib’s benefit in GIST, the program expanded in 2002 to support patients with GIST. To evaluate the possible impact of these access programs, we analyzed the demographic characteristics, treatment duration, and survival of patients who received imatinib and/or sunitinib for GIST through GIPAP and MAS.

## Methods

This cohort study received a waiver for human participant review by the University of Washington Institutional Review Board, and the need for informed consent was also waived. We followed the Strengthening the Reporting of Observational Studies in Epidemiology (STROBE) guideline for cohort studies.

### Patient Cohort

The cohort included patients enrolled in 2 treatment access programs administered by The Max Foundation: GIPAP (January 1, 2001, to December 31, 2016) and MAS (January 1, 2017, to October 12, 2020). Approved indications for imatinib included adjuvant therapy in high-risk disease identified by pathologic evaluation of resected tumor or biopsy-proven unresectable or metastatic GIST. All patients were reported by treating physicians to be positive for CD117(c-kit). All patients with an approved indication were treated with imatinib in the first-line setting. Since 2015, physicians in participating countries have been able to request sunitinib as second-line treatment.

### Data Collection

Data were collected for the purpose of verifying that individual case requests fulfilled program criteria, planning and forecasting, and assessing the need for patient support. Local treating physicians submitted demographic data for participating patients, including confirmation of high-risk disease by pathologic evaluation of resected tumor or biopsy-proven unresectable or metastatic GIST, age at approval into the access program, sex, and country of origin; doses administered; and reasons for and date of treatment discontinuation. Within the program guidelines, The Max Foundation requested an update every 3 to 4 months from each treating physician regarding the need for additional treatment of each enrolled patient. We further classified patients based on World Bank income groups by country^23^ and country-specific life expectancies.^24^ We also calculated patient contacts per year with the local program team from The Max Foundation. Reporting race and ethnicity were not required for participation in The Max Foundation programs and were therefore not included in the analysis.

### Censoring Rules

Patients were censored for OS analysis if they did not have a death date and for time to treatment discontinuation (TTD) analysis if they did not have a date of treatment discontinuation due to an event. For metastatic or unresectable disease, reasons for discontinuation that were censored included patients who obtained alternative access to treatment (3433 patients [34.8%] receiving imatinib), patients who stopped for unknown reasons but were known to be alive (428 [4.3%]), patients who refused treatment (27 [0.3%]), and patients who discontinued treatment due to pregnancy (1 [0.01%]). Patients who were lost to follow-up (LTFU) for other reasons were presumed deceased. For patients treated in the adjuvant setting, reasons for discontinuation that were censored included patients who obtained alternative access to treatment (478 [22.8%] of population), patients who stopped for unknown reasons but were known to be alive (528 [25.1%]), and patients who discontinued treatment due to pregnancy (2 [0.1%]).

### Statistical Analysis

Data were analyzed from October 13, 2020, to January 30, 2024. In the cohort with metastatic or unresectable disease, patients who were LTFU were either censored as per the criteria above or presumed deceased at treatment discontinuation. Kaplan-Meier analysis was used to estimate the distribution of TTD and OS. Reasons for discontinuation included “patient has passed away,” “adverse event,” “clinical reason,” and “patient not responding to treatment.” In the cohort of patients treated in the adjuvant setting, both a standard censoring and an imputation-based informed censoring model (eMethods in Supplement 1) using the Informative Censoring R package, version 4.2.2 (R Project for Statistical Computing) was applied to estimate event times for patients who were LTFU.^25^

Cox proportional hazards modeling was used to evaluate the effect of the following variables on each outcome: sex, age at approval for enrollment into The Max Foundation program, World Bank country income classification, and frequency of The Max Foundation team patient contact by quartile as categorical variables. Age was treated as a continuous variable. The same methods were applied to conduct subgroup analyses of TTD and OS in imatinib-treated patients by setting (adjuvant and unresectable or metastatic disease) according to the following categories of age at diagnosis: younger than 40, 40 to 55, 56 to 65, and older than 65 years. Two-sided *P* < .05 indicated statistical significance.

## Results

### Cohort Demographics

**Figure 1.**
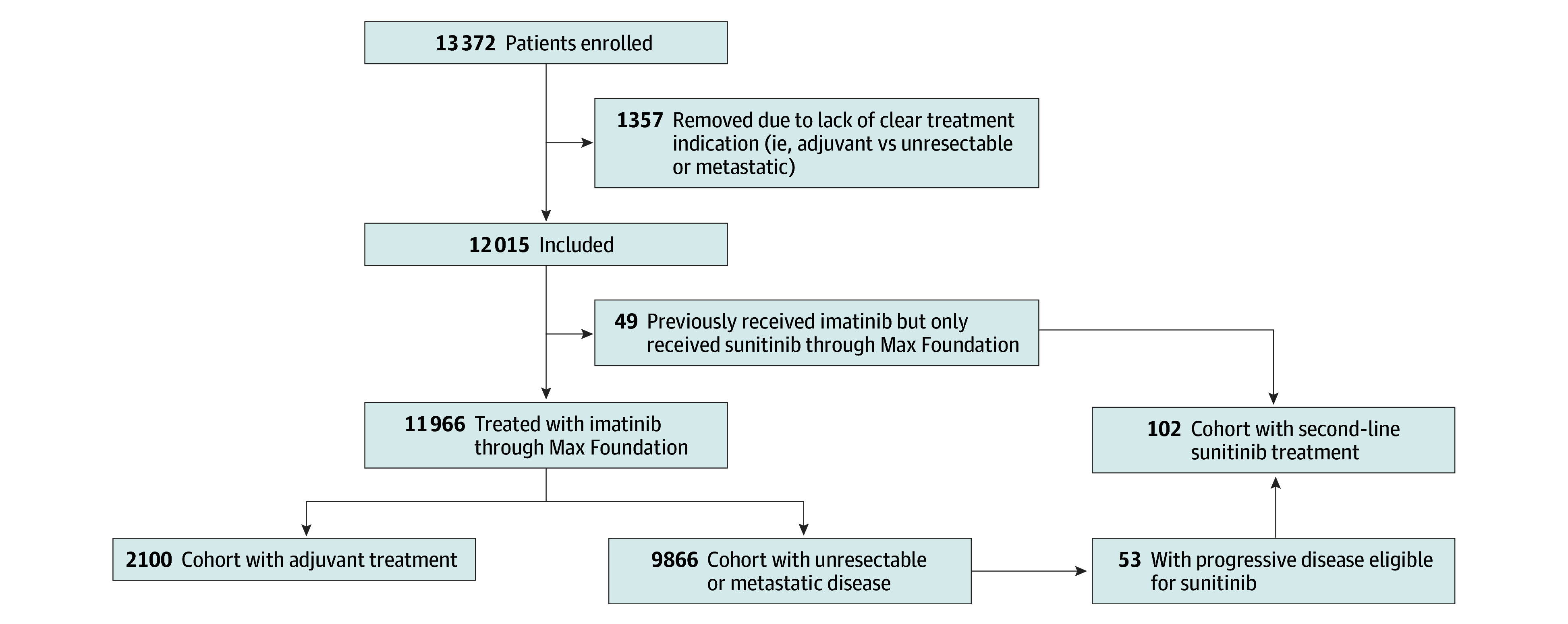
Patient Cohort Schema

**Figure 2.**
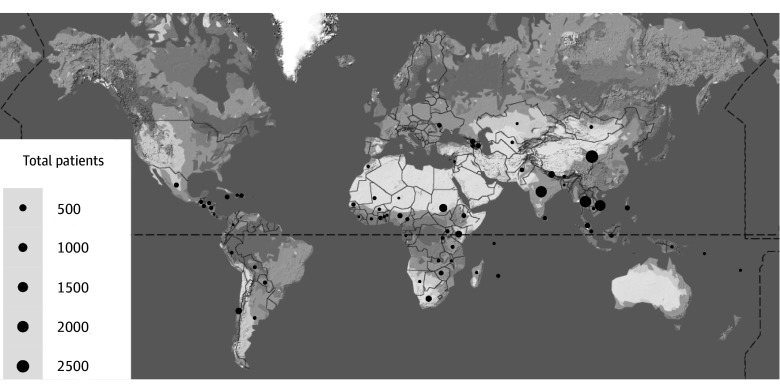
Global Patient Distribution The number of patients in each country are shown in the bubble plot with size of circle proportional to the number of patients administered treatment through The Max Foundation programs.

The combined cohort of programs administered by The Max Foundation included 13 372 patients treated for GIST. Of these, 1357 patients were excluded due to lack of clear treatment indication (adjuvant treatment or unresectable or metastatic disease). The final cohort included 12 015 unique patients (5076 female [42.4%] and 6890 male [57.6%]; median age, 54 [range, 0-100] years; 627 [5.2%] from low-income countries). Of these, 2100 patients were treated with imatinib in the adjuvant setting and 9866 were treated with imatinib for metastatic or unresectable disease. The median follow-up was 979 (range, 1-3924) days for those treated with adjuvant imatinib and 793 (range, 1-6732) days for those treated for metastatic or unresectable disease. The sunitinib cohort of 102 patients (median follow-up, 449 [range, 1-1807] days) included 53 patients already included in the cohort receiving imatinib for unresectable or metastatic disease and 49 patients who received imatinib from another source and were only included in the sunitinib analysis (Figure 1). Patients were from 66 countries (Figure 2 and eTable 1 in Supplement 1). The median age was 54 (range, 8-88) years in the adjuvant imatinib group and 55 (range, 0-100) years in the group with unresectable or metastatic GIST. Male patients constituted 1023 of 2100 patients (48.7%) in the group receiving adjuvant imatinib and 5867 of 9866 patients (59.5%) in the group with metastatic or unresectable GIST. Most patients were from countries classified as lower-middle or upper-middle income (Table and eTable 2 in Supplement 1).

**Table.** Demographic and Dosing Data for Imatinib-Treated Patients

Characteristic	Treatment indication^a^		
	Adjuvant (n = 2100)	Unresectable or metastatic disease (n = 9866)	Overall (n = 11 966)
Sex			
Female	1077 (51.3)	3999 (40.5)	5076 (42.4)
Male	1023 (48.7)	5867 (59.5)	6890 (57.6)
Age at approval, median (range), y	54 (8-88)	55 (0-100)	54 (0-100)
World Bank income group			
Low	32 (1.5)	595 (6.0)	627 (5.2)
Lower middle	1685 (80.2)	3495 (35.4)	5180 (43.3)
Upper middle	240 (11.4)	5537 (56.1)	5777 (48.3)
High	143 (6.8)	239 (2.4)	382 (3.2)
Minimum imatinib dose, mg/d			
100	0	2 (0.02)	2 (0.02)
200	2 (0.1)	41 (0.4)	43 (0.4)
300	48 (2.3)	259 (2.6)	307 (2.6)
400	2050 (97.6)	9413 (95.4)	11 463 (95.8)
600	0	136 (1.4)	136 (1.1)
800	0	15 (0.2)	15 (0.1)
Maximum imatinib dose, mg^b^			
200	0	3 (0.03)	3 (0.03)
300	0	24 (0.2)	24 (0.2)
400	2084 (99.2)	7704 (78.1)	9788 (81.8)
600	8 (0.4)	1217 (12.3)	1225 (10.2)
800	8 (0.4)	874 (8.9)	882 (7.4)

^a^ Unless otherwise indicated, data are expressed as No. (%) of patients.

^b^ Forty-four patients did not have a maximum dose clearly reported and were not included in this analysis.

### Treatment

Most patients receiving imatinib mesylate (11 463 [95.8%]) were initially prescribed 400 mg/d. Most patients receiving sunitinib malate (99 of 102 [97.1%]) were prescribed 50 mg for 4 weeks, followed by 2 weeks without the therapy (Table and eTable 2 in Supplement 1). Physicians were requested to report dose adjustments, but this reporting was not required to maintain patients in the access program.

### Outcomes for Patients With Metastatic or Unresectable GIST

**Figure 3.**
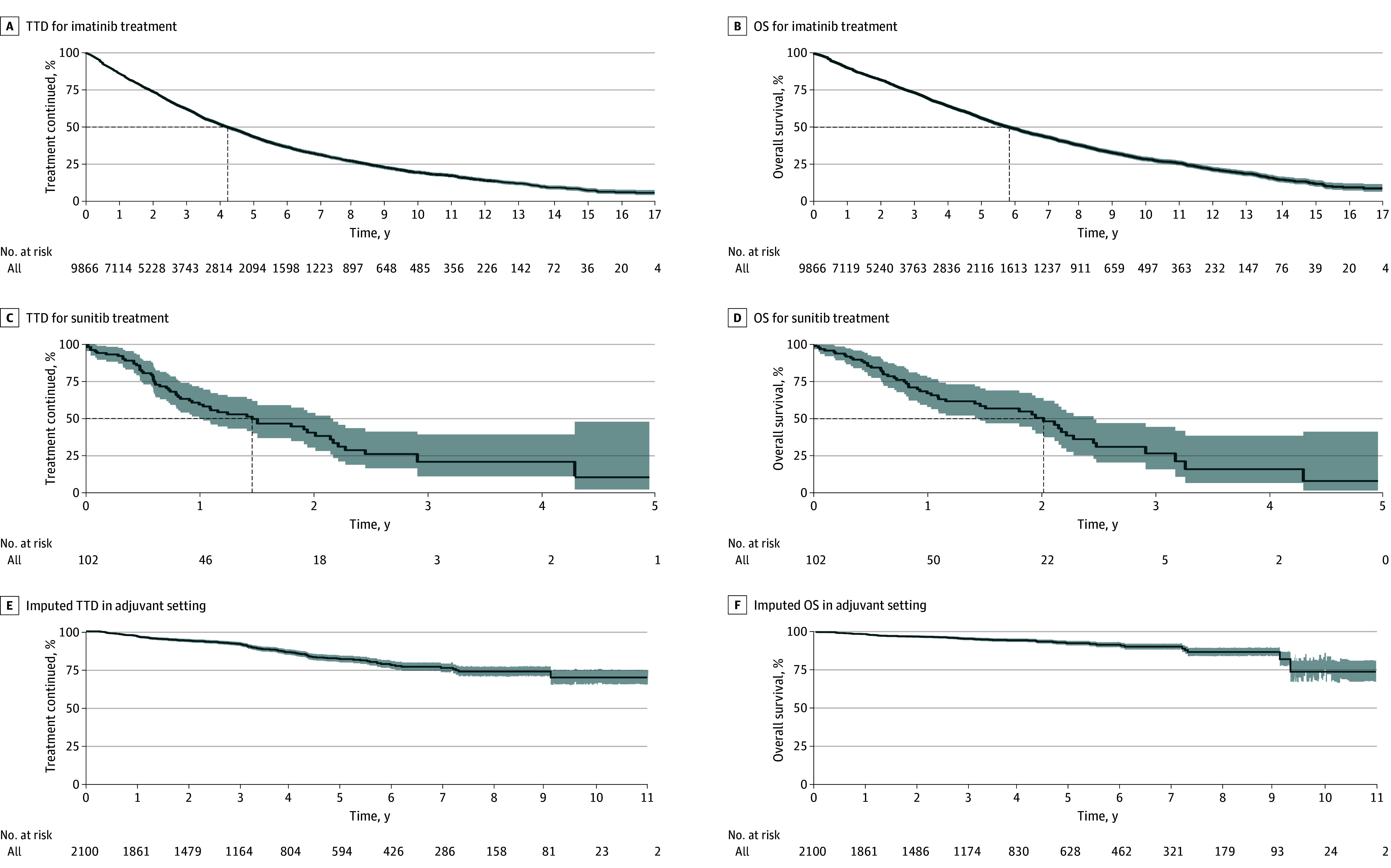
Time to Treatment Discontinuation (TTD) and Overall Survival (OS) Kaplan-Meier curves for TTD and OS are shown for patients treated with imatinib in the metastatic or unresectable setting (A and B) and patients treated with sunitinib following progression during first-line therapy (C and D). Imputed TTD and OS are shown for patients treated with imatinib in the adjuvant setting. If reached, median TTD and OS is indicated by dashed line. The shading along the curves indicates the 95% CIs.

A total of 2282 patients (23.1%) patients in the cohort with metastatic or unresectable GIST were LTFU. Standard censoring resulted in a median OS of nearly 15 years (eFigure 1 in Supplement 1). Following presumption of death for patients who were LTFU, patients treated with imatinib for unresectable or metastatic disease had a median TTD of 4.2 (95% CI, 4.1-4.4) years (Figure 3A) and median OS of 5.8 (95% CI, 5.6-6.1) years (Figure 3B). Among patients treated with second-line sunitinib, median TTD was 1.5 (95% CI, 1.0-2.1) years (Figure 3C) and median OS was 2.0 (95% CI, 1.5-2.5) years (Figure 3D).

### Outcomes for Patients Treated With Adjuvant Imatinib

A total of 2100 patients were treated with adjuvant imatinib and, of these, 473 (22.5%) were LTFU. Using standard censoring of patients who were LTFU resulted in a 10-year treatment discontinuation rate of 32.2% (95% CI, 20.4%-42.3%) and a 10-year OS rate of 77.1% (95% CI, 63.8%-93.2%) (eFigure 2A and B in Supplement 1). We then performed a sensitivity analysis to determine the extreme assumption that all patients who were LTFU were deceased, as for patients with metastatic or unresectable GIST. This increased the 10-year treatment discontinuation rate to 67.5% (95% CI, 60.0%-73.6%) and decreased 10-year OS to 31.7% (95% CI, 23.6%-42.5%) (eFigure 2C and D in Supplement 1). However, using an informed censoring model, patients treated with imatinib in the adjuvant setting had an imputed 10-year treatment discontinuation rate of 30.4% (95% CI, 25.6%-34.9%) (Figure 3E) and 10-year OS rate of 73.8% (95% CI, 67.2%-81.1%) (Figure 3F). The stability of the model was tested by running multiple gamma values. Alterations in the gamma value of 1.0 indicated a clinical inference that patients who were LTFU would be 10.0 times more likely to have an event than similar patients in the same cohort, resulting in a decreased mean (SD) 10-year OS rate after 20 imputations of 60.8% (9.7%). Gamma values of 1.5 and 2.0 were 31.6 and 100.0 times more likely, respectively, to have an event than similar patients in the same cohort, resulting in mean (SD) 10-year OS rates of 58.6% (11.0%) and 48.0% (12.0%), respectively (eTable 3 in Supplement 1).

### Multivariate Analysis

**Figure 4.**
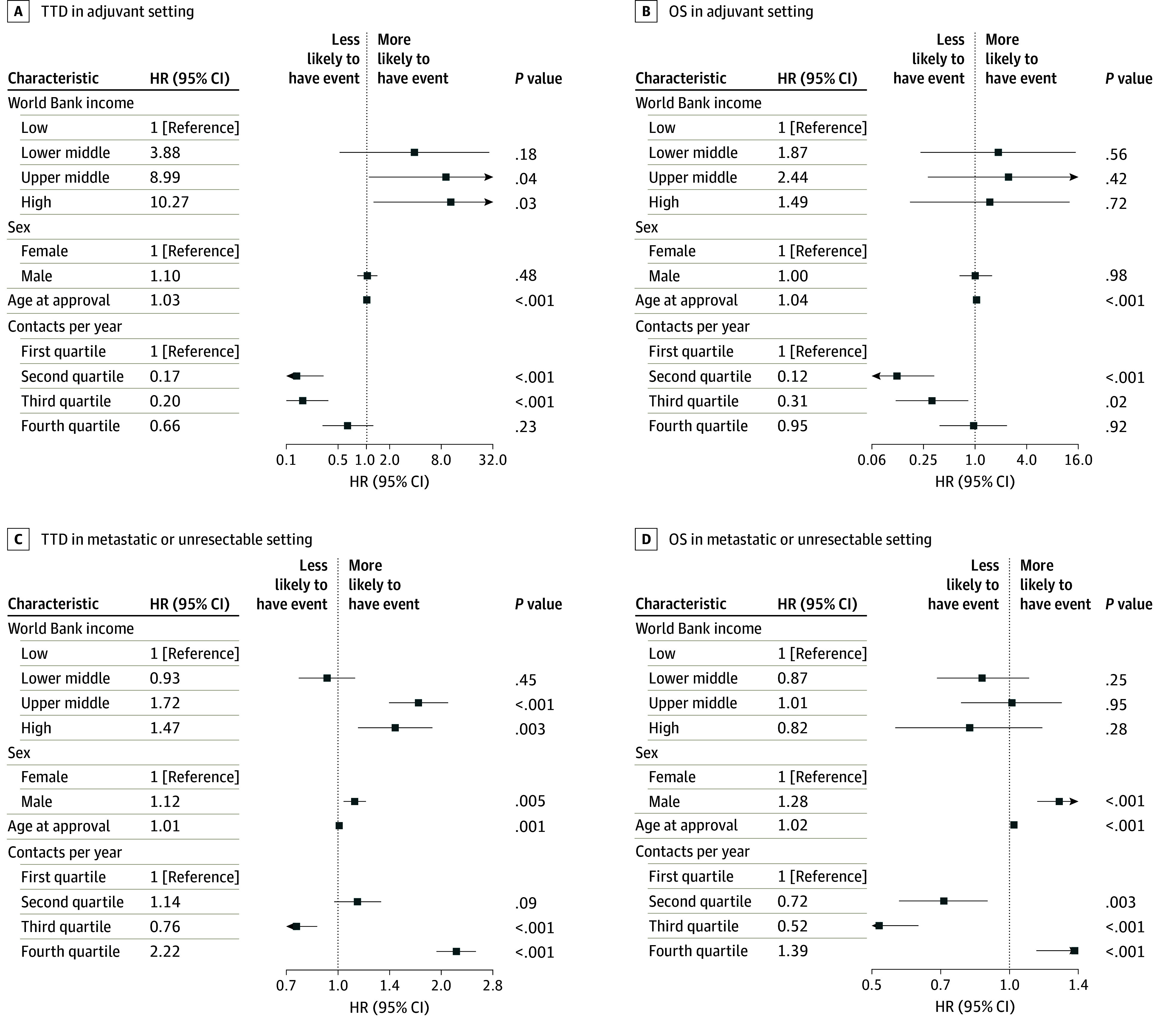
Multivariate Analysis of Outcomes Time to treatment discontinuation (TTD) and overall survival (OS) are shown for cohorts in the adjuvant metastatic or unresectable settings. Hazard ratios (HRs) are calculated based on ratio to reference group within each covariate.

There was no apparent association based on sex in the adjuvant setting (Figure 4A and B). However, male sex was a negative prognostic factor associated with TTD (HR, 1.12 [95% CI, 1.03-1.20]; *P* = .005) and OS (HR, 1.28 [95% CI, 1.15-1.43]; *P* < .001) in the cohort with metastatic or unresectable GIST (Figure 4C and D). Age as a continuous variable was a significant negative predictive factor associated with TTD and OS in all cohorts (Figure 4). While there were notable differences among income groups and the frequency of contact quartiles, there was no clear association between increasing or decreasing income or frequency of contact (eFigure 3A-D in Supplement 1).

### TTD Stratified by Age and Sex Subgroups in Imatinib-Treated Patients

Patients 50 years or older in the cohort with metastatic or unresectable GIST had a significantly worse TTD compared with patients younger than 50 years (HR, 1.25 [95% CI, 1.15-1.35]; *P* < .001). In the adjuvant cohort, differences were more pronounced (HR, 2.01 (95% CI, 1.47-2.75]; *P* < .001). Time to treatment discontinuation by sex was not significantly different for patients younger than 50 years (HR, 0.87 [95% CI, 0.47-1.60]; *P* = .65). However, for patients 50 years or older, male patients had a significantly shorter TTD (HR, 2.26 [95% CI, 1.45-3.50]; *P* < .001). We further stratified our cohort by age at approval for the access program into 4 age groups (<40, 40-55, 56-65, and >65 years). We found that in patients with metastatic or unresectable GIST, there was no notable difference between those younger than 40 and aged 40 to 55 years; however, the TTD was significantly shorter for patients aged 56 to 65 years (HR, 1.17 [95% CI, 1.03-1.32]; *P* = .01) and older than >65 years (HR, 1.55 [95% CI, 1.36-1.76]; *P* < .001). In the adjuvant setting, compared with patients younger than 40 years, the TTD was significantly different for patients aged 40 to 55 years (HR, 1.90 [95% CI, 1.09-3.30]; *P* = .007), aged 55 to 65 years (HR, 2.35 [95% CI, 1.31-4.22]; *P* < .001), and older than 65 years (HR, 4.22 [95% CI, 2.39-7.45]; *P* < .001) (eTable 4 in Supplement 1).

### Life Expectancy and Age of Approval

There was no correlation between age at time of drug access approval and life expectancy by country in either the adjuvant (*R* = −0.26; *P* = .10) or metastatic cohort (*R* = 0.05; *P* = .68). Further details are found in eFigure 3 in Supplement 1.

## Discussion

To our knowledge, this study represents the largest evaluation of outcomes for patients treated with imatinib for GIST and is the first to include patients predominantly from LMICs. Our findings suggest that administering cancer treatment orally may be feasible across a large network of local treating physicians in LMICs, with outcomes comparable to those reported in clinical trials and data in mostly North American and European populations. In the group with metastatic or unresectable GIST, despite other potential barriers and conservative death estimates, overall survival was 5.8 years (imatinib, first-line) and 2.0 years (sunitinib, second-line) compared with approximately 4.6 years and 1.4 years, respectively, reported in higher-resource settings.^5,17^ When we analyzed subgroups of patients based on World Bank incomes by the patient’s country of residence, there was no significant difference in outcomes. This underscores the importance of access to appropriate cancer therapy in LMICs.

According to the World Bank, 75% of the world’s population lives in middle-income countries and 9% of the world’s population lives in low-income countries.^23,26^ While not an exact match, our population includes 5.2% from low-income countries and reflects the overall global distribution in the setting of constraints to opening the access program in certain countries for various reasons. These include social or political barriers, and capacity to diagnose and treat patients with GIST, that might account for the slightly lower number of participants from low-income countries.

This cohort was younger by about 10 years at the time of GIST diagnosis compared with those in previously reported GIST studies.^10,11,12,27^ Rather than the actual date of diagnosis, we used the date of drug approval by The Max Foundation to determine age because this was more reliable across the dataset. This procedure strengthens this finding, as any bias would favor an older cohort compared with cohorts that used age at diagnosis. It is uncertain from our data whether biological differences or wider systemic variables explain the younger median age in our cohort. There are several potential explanations for why this cohort is younger than other reported cohorts with GIST. First, the mean life expectancy by country in this predominantly LMIC cohort is lower than that of prior cohorts with GIST in high-resource settings. However, we were unable to show that age at the time of imatinib approval for either adjuvant or unresectable disease correlated with life expectancy. Factors such as a potential hesitancy or inability to seek care among the elderly and resource limitations impeding access to clinical care may also affect the overall age of our cohort. Of note, patients in this program for the treatment of CML were younger at diagnosis than in historical cohorts.^28^ Based on the available data, we can only speculate as to the reasons for why our cohort is younger than similar cohorts reported in high-income settings, but we believe this finding warrants further investigation.

The younger age of patients with GIST in our study may also contribute to the longer overall survival of patients in the cohort with unresectable or metastatic disease compared with prior studies. However, when we stratified patients by age group, there was a modest decrease in TTD for older patients in the cohort with unresectable or metastatic cohort, but this was unlikely to be clinically meaningful. An alternative explanation for improved outcomes in the cohort with unresectable or metastatic GIST is that access to higher-risk surgical procedures may have been limited. Consequently, relatively less aggressive tumors may have been deemed unresectable. Furthermore, a lack of access to advanced imaging and endoscopy in resource-limited settings may result in delayed diagnosis of a biologically less aggressive tumor until it is at a stage that is considered unresectable. However, this potential lack of early detection makes our finding of such a drastically earlier age at diagnosis even more intriguing. We found that younger age was associated with a longer TTD. While an imperfect comparison, our findings largely agree with the finding of worse outcomes in patients 50 years or older, given that nearly 90% of that cohort was treated in the adjuvant setting.^13^ The 2 major clinical trials of imatinib for metastatic or unresectable disease^5,7^ demonstrated an improved OS for younger patients by decade of diagnosis, but this difference was not reflected in PFS, suggesting competing risks of death. Also in agreement with these studies is our findings of male sex as a negative prognostic marker in patients with metastatic or unresectable disease; however, we demonstrated that this was not an independent prognostic variable in the adjuvant cohort. We further explored the latter finding by stratifying patients both by age and sex and found that for patients 50 years or older, female patients had a longer TTD compared with male patients 50 years or older, which contrasts with the work of Kramer et al,^13^ who found significantly better disease-specific survival in female vs male patients younger than 50 years, but no difference in patients 50 years or older.

We also noted that there were very few dose decreases reported in the imatinib cohort compared with prior reports in which 16% to 25% of patients receiving imatinib at standard dosing required at least 1 dose modification or change.^5,8,14^ This may be partially explained by the relatively younger age of patients receiving treatment and limitations in data collection, including unreported dose reductions. We also used country-level income data, which does not reflect the resources of an individual patient or countries with large income inequality, which may influence outcomes.

All program participants were assessed for c-kit protein expression; however, screening for specific *KIT* or *PDGFRA* sequence variants, recommended when feasible per treatment guidelines in high-income countries and important for identifying the subset of patients whose tumors may not be sensitive to imatinib,^29,30^ is not a prerequisite for participation in the drug access program. Making sequence variant testing both affordable and accessible could prevent a subset of patients with GIST from undergoing potentially ineffective treatments and avoid unnecessary adverse effects. Nonetheless, such testing relies on resource-intensive sequencing diagnostics that may not be readily accessible in the LMIC settings from which the cohort population is derived.^31^ Unfortunately, there is a gap in research assessing barriers to implementation and cost-effectiveness of sequencing diagnostics for cancer care in LMIC.^32^ As sequencing approaches become less expensive and health systems evolve to incorporate the infrastructure and staffing required to perform this testing, processing of GIST samples for sequence variant testing may be more possible in the future.

### Limitations

This analysis has some limitations. The primary objective of The Max Foundation’s programs is to provide access to treatment rather than researching treatment outcomes and survival. Therefore, a careful balance of data collection for monitoring and evaluation must be weighed against the potential burden on a strained health care system and the patients’ desire to share further data. While clinical trials and cohorts in high-income countries can uniformly define markers of progression through strict imaging schedules, access to imaging is variable across LMICs, thus preventing direct measurement of PFS.^33^ We used TTD as a surrogate outcome measure, since treatment start and end dates were reliably reported. This included progression events but may have also reflected other reasons for discontinuation, including drug intolerance or patient and/or clinician preference. Additionally, in some cases, physicians may have continued imatinib treatment beyond progression when access to subsequent therapy was limited, which would further complicate direct comparisons of PFS to TTD. As in our cohort, the analysis of outcomes across a range of disease types is complicated in lower-resource health care systems by a large percentage of patients who are LTFU.^34^ However, simply ignoring these populations results in inequitable representation for most of the global population in longitudinal studies. We attempted to mitigate the impact of patients who are LTFU in our cohort by using an imputation-based modeling approach for patients treated in the adjuvant setting. However, we cannot know for certain that these patients would have had outcomes that are better or worse than those who were not LTFU. Despite these limitations, this approach does offer a reasonable alternative for large heterogenous studies in which additional follow-up to fill in missing data is not possible. In contrast to the adjuvant treatment of resected, high-risk GIST, a cure in patients with unresectable or metastatic GIST is highly unlikely. Therefore, we chose a conservative approach and presumed patients who were LTFU were deceased. Finally, our analysis is susceptible to selection bias, as patients had to reach physicians who were aware of and willing to access treatment for their patients through The Max Foundation. We also do not have data on the incidence of GIST in most LMICs or the frequency of patients who may have presented to their physician and declined treatment. Finally, when evaluating frequency of contact as a covariate, patient-physician interactions have been driven both by improved disease monitoring and patient illness, thereby masking any related signal.

## Conclusions

In this cohort study of patients with GIST predominantly from LMICs who received orally administered therapy, patients had clinical outcomes similar to those observed in high-resource countries. In addition to demonstrating the efficacy of imatinib and sunitinib for adjuvant treatment and for metastatic or unresectable GIST in a global population, our analysis makes clear that delivering efficacious cancer therapy in the LMIC setting was possible. We also highlighted the importance of partnering with clinicians who treat cancer in LMICs to deliver efficacious cancer therapy to achieve equitable cancer outcomes. While imatinib is an ideal candidate, given the simplicity of administration and relative tolerability, other orally administered cancer drugs are available. Specifically, cancers such as breast (selective estrogen receptor modulators, aromatase inhibitors, CDK4/6 inhibitors), prostate (androgen biosynthesis inhibitors), and lung (TKIs) have highly effective oral therapies. Unfortunately, many drugs are currently priced beyond availability for most of the world’s patients with cancer. Our findings suggest that delivering orally administered anticancer agents is feasible and sustainable in LMICs and highlight the potential for drug access programs to save lives.

## Notes

Supplement 1. **eMethods.** Statistical Methods, Informed Censoring Model, and Sensitivity Analysis **eTable 1.** Frequency of Unique Patients by Country of Treatment **eTable 2**. Demographic and Dosing Data for Sunitinib-Treated Cohort **eTable 3.** Overall Survival (OS) Outcomes Based Upon Varied Assumptions and Adjusted Gamma Values in an Imputation-Based Modeling Approach for Patients Treated With Imatinib in the Adjuvant Setting **eTable 4.** Hazard Ratios of Outcomes Stratified by Age at Approval **eFigure 1.** Kaplan-Meier Curve of Overall Survival (OS) in the Metastatic or Unresectable Cohort Using Standard Censoring of Patients Lost to Follow-Up (LTFU) **eFigure 2.** Sensitivity Analysis **eFigure 3.** Pearson Correlation Test of Country Life Expectancy vs Age at Imatinib Approval

Supplement 2.
Data Sharing Statement
