# Supplementary material for: Treatment Access for Gastrointestinal Stromal Tumor in Predominantly Low- and Middle-Income Countries
Source: JAMA Netw Open. 2024 Apr 3;7(4):e244898. doi: 10.1001/jamanetworkopen.2024.4898 (PMC10993077; doi:10.1001/jamanetworkopen.2024.4898)
Supplement: Supplement 1. — eMethods. Statistical Methods, Informed Censoring Model, and Sensitivity Analysis eTable 1. Frequency of Unique Patients by Country of Treatment eTable 2. Demographic and Dosing Data for Sunitinib-Treated Cohort eTable 3. Overall Survival (OS) Outcomes Based Upon Varied Assumptions and Adjusted Gamma Values in an Imputation-Based Modeling Approach for Patients Treated With Imatinib in the Adjuvant Setting eTable 4. Hazard Ratios of Outcomes Stratified by Age at Approval eFigure 1. Kaplan-Meier Curve of Overall Survival (OS) in the Metastatic or Unresectable Cohort Using Standard Censoring of Patients Lost to Follow-Up (LTFU) eFigure 2. Sensitivity Analysis eFigure 3. Pearson Correlation Test of Country Life Expectancy vs Age at Imatinib Approval [file jamanetwopen-e244898-s001.pdf]

Supplementary Online Content

Briercheck EL, Wrigglesworth JM. Garcia-Gonzalez I, et al. Treatment access for gastrointestinal stromal tumor in predominantly low- and middle-income countries. *JAMA Netw Open*. 2024;7(4):e244898. doi:10.1001/jamanetworkopen.2024.4898

eMethods. Statistical Methods, Informed Censoring Model, and Sensitivity Analysis
eTable 1. Frequency of Unique Patients by Country of Treatment
eTable 2. Demographic and Dosing Data for Sunitinib-Treated Cohort
eTable 3. Overall Survival (OS) Outcomes Based Upon Varied Assumptions and Adjusted Gamma Values in an Imputation-Based Modeling Approach for Patients Treated With Imatinib in the Adjuvant Setting
eTable 4. Hazard Ratios of Outcomes Stratified by Age at Approval
eFigure 1. Kaplan-Meier Curve of Overall Survival (OS) in the Metastatic or Unresectable Cohort Using Standard Censoring of Patients Lost to Follow-Up (LTFU)
eFigure 2. Sensitivity Analysis
eFigure 3. Pearson Correlation Test of Country Life Expectancy vs Age at Imatinib Approval

This supplementary material has been provided by the authors to give readers additional information about their work.

© 2024 Briercheck EL et al. *JAMA Network Open*.

eMethods. Statistical Methods, Informed Censoring Model, and Sensitivity Analysis

For imputation-based informed censoring missing outcomes were imputed via a bootstrap sampling of outcomes in patients with known outcomes. This approach uses common metadata between patients with known outcomes and LTFU patients to predict the most likely outcome in LTFU cases. Demographic data used to match patients with known outcomes and those LTFU were age at approval, sex, and income classification. After 20 iterations, the resulting datasets were used to create the Cox models and Kaplan Meier plots. A gamma value can be set to further adjust the model based upon whether patients LTFU are predicted by clinical inference to be more or less likely to have an event than the known cohort. For our primary analysis we chose an agnostic approach and set this value to zero (indicates no presumed difference in outcome between known and LTFU group). Stability of the model and sensitivity analysis was done by altering the gamma value 0, 0.5, 1.0, 1.5 and 2.0.

Data analysis and visualization were conducted using several R packages, including InformativeCensoring (v0.3.5), dplyr (v1.0.9), ggmap (v3.0.0), ggplot2 (v3.3.6), survminer (v0.4.9), tableone (v0.13.2), forestplot (v3.1.3), and R v4.1 or v4.2 packages, which are tools for data manipulation, plotting, and statistical analysis.

A p-value <0.05 was considered statistically significant.

© 2024 Briercheck EL et al. *JAMA Network Open*.

eTable 1. Frequency of Unique Patients by Country of Treatment

Country	Total Patients	Country	Total Patients	Country	Total Patients
Argentina	19	El Salvador	32	Madagascar	10
Armenia	26	Ethiopia	46	Malawi	<5
Azerbaijan	74	Fiji	<5	Malaysia	166
Benin	<5	Gabon	<5	Mali	21
Bhutan	<5	Georgia	107	Mauritius	37
Bolivia	31	Ghana	41	Mexico	141
Burkina Faso	7	Guatemala	64	Moldova	53
Cambodia	14	Haiti	9	Mongolia	14
Cameroon	24	Honduras	41	Morocco	18
Chile	333	India	1874	Namibia	<5
China	2534	Indonesia	69	Nepal	238
Colombia	<5	Jamaica	76	Nicaragua	33
Costa Rica	<5	Kazakhstan	<5	Niger	<5
Cote d'Ivoire	6	Kenya	445	Nigeria	89
Dominican Republic	72	Lebanon	<5	Pakistan	113

© 2024 Briercheck EL et al. *JAMA Network Open*.

Papua New Guinea	<5	Sierra Leone	<5	Thailand	2018
Paraguay	33	Singapore	41	Togo	<5
Peru	16	Solomon Islands	<5	Uganda	42
Philippines	145	South Africa	324	Uzbekistan	9
Rwanda	8	Sri Lanka	55	Vietnam	1714
Senegal	57	Sudan	498	Zambia	24
Seychelles	8	Tanzania	23	Zimbabwe	86

© 2024 Briercheck EL et al. *JAMA Network Open*.

eTable 2. Demographic and Dosing Data for Sunitinib-Treated Cohort

	**Overall (N=102)**
**Sex**	
F	44 (43.1%)
M	58 (56.9%)
**Age at approval**	
Median [Min, Max]	51.0 [24.0, 76.0]
**World Bank Income Group**	
Low income	33 (32.4%)
Lower-middle income	63 (61.8%)
Upper-middle income	6 (5.9%)
High-income	0 (0%)
**Minimum sunitinib dose (mg)**	
25	3 (2.9%)
37	3 (2.9%)
50	96 (94.1%)
**Maximum sunitinib dose (mg)**	
25	1 (1.0%)
50	99 (97.1%)
missing	2 (2.0%)

© 2024 Briercheck EL et al. *JAMA Network Open*.

eTable 3. Overall Survival (OS) Outcomes Based Upon Varied Assumptions and Adjusted Gamma Values in an Imputation-Based Modeling Approach for Patients Treated With Imatinib in the Adjuvant Setting

	Events	Median OS in years (SD)	3-year % OS (SD)	5-year % OS (SD)	10-year % OS (SD)
EventOS	92.00	NA	95.6	92.8	77.1
EventLTFOS	569.00	7.012	76.7	58.6	31.7
	**Average Events (SD)**	**Median OS in years (SD)**	**3-year Average % OS (SD)**	**5-year Average % OS (SD)**	**10-year Average % OS (SD)**
Gamma=0	137.55 (11.5)	NA	95.6 (0.2)	92.5 (0.6)	73.8 (8.1)
Gamma=0.5	158.50 (17.7)	NA	95.2 (0.2)	91.5 (1.1)	69.8 (8.9)
Gamma=1	184.50 (16.4)	9.306 (<0.1)	94.8 (0.4)	89.7 (1.1)	60.8 (9.7)
Gamma=1.5	209.1 (23.4)	9.306 (<0.1)	94.3 (0.5)	88.5 (1.4)	58.6 (11.0)
Gamma=2	266.60 (29.0.1)	9.202 (0.1)	93.2 (0.7)	84.4 (2.6)	48.0 (12.0)

EventOS = standard censoring. EventLTFOS= all lost to follow-up cases are presumed deceased. Subsequent rows indicate imputation-based averages of 20 imputations with adjusted gamma values as indicated. SD = standard deviation, NA= not applicable

© 2024 Briercheck EL et al. *JAMA Network Open*.

eTable 4. Hazard Ratios of Outcomes Stratified by Age at Approval

**Stratification**	**Adjuvant**	**Unresectable/Metastatic**
**Two Group Age at approval**	**Hazard Estimate**	**Hazard Estimate**
<50	1.0	1.0
≥50	2.01[1.5,2.7] (p<.001)	1.25 [1.2,1.3] (p<.001)
**Sex and Two Group Age at approval**		
Female, <50	1.0	1.0
Female, ≥50	2.1 [1.0,4.3] (p=.06)	1.2 [1.1,1.4,] (p=.001)
Male, <50	0.87 [0.5,1.6] (p=.65)	1.1 [0.9,1.2] (p=.305)
Male, ≥50	2.3 [1.5,3.5] (p<.001)	1.4 [1.2,1.5] (p<.001)
**Four Group Age at approval**		
<40	1.0	1.0
≥40-<55	1.9 [1.1,3.3] (p=.024)	1.0 [0.9,1.1] (p=.783)
≥55-65	2.4 [1.3,4.2] (p=.004)	1.2 [1.0,1.3] (p=.014)
≥65	4.2 [2.4,7.5] (p<.001)	1.5 [1.4,1.8] (p<.001)

Hazard ratios of outcomes stratified by age at approval in two groups, two age groups at approval further stratified by sex and four age groups at approval. Ninety-five percent confidence intervals are shown in brackets.

© 2024 Briercheck EL et al. *JAMA Network Open*.

eFigure 1. Kaplan-Meier Curve of Overall Survival (OS) in the Metastatic or Unresectable Cohort Using Standard Censoring of Patients Lost to Follow-Up (LTFU)

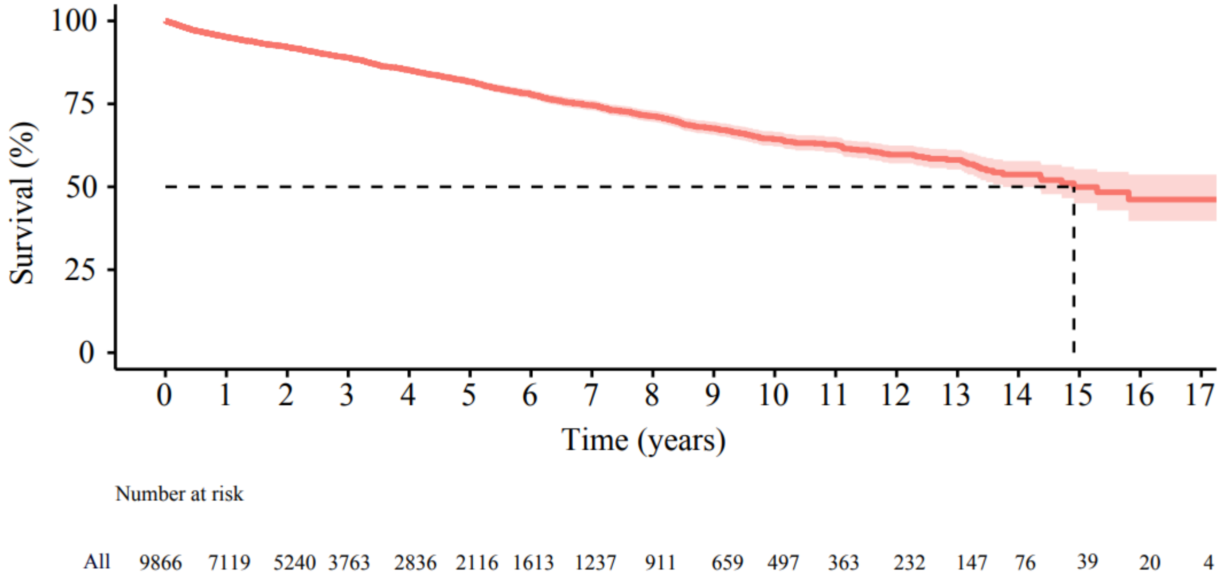

Dashed line demonstrates median OS. Number at risk at each time point are shown immediately below each curve. Confidence intervals are indicated by shading along the curve.

© 2024 Briercheck EL et al. *JAMA Network Open*.

eFigure 2. Sensitivity Analysis

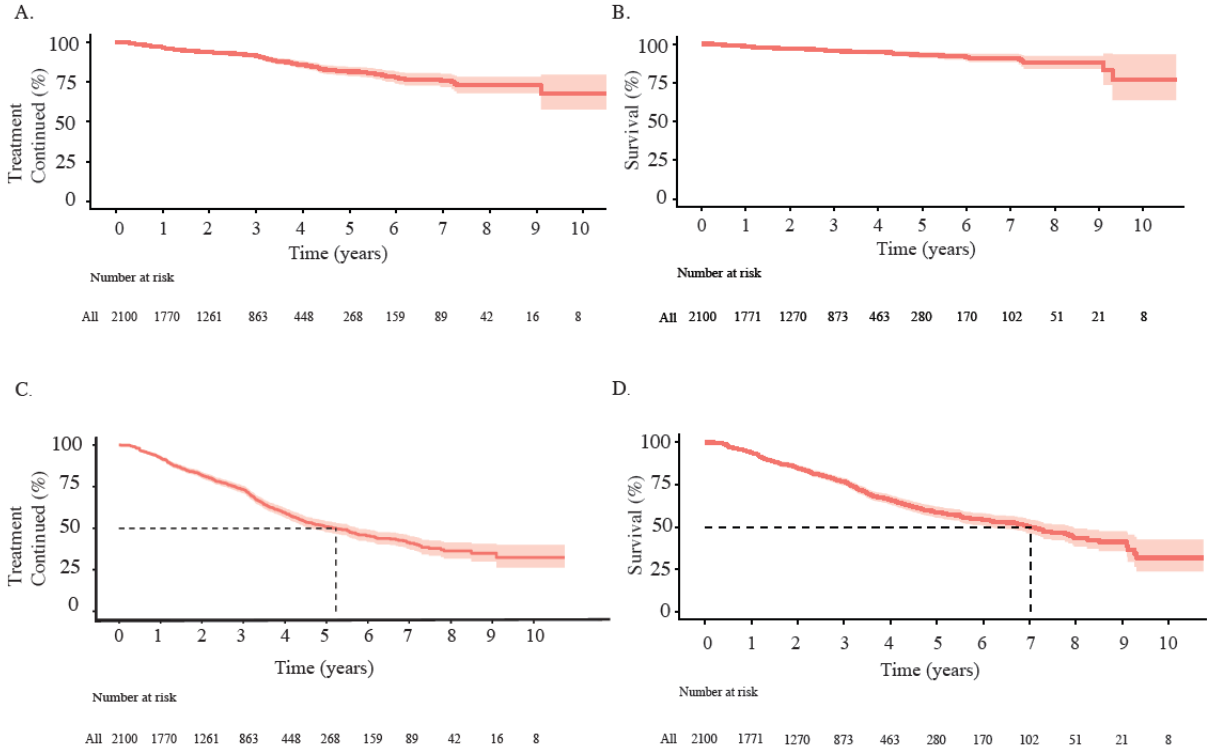

Kaplan-Meier curves of time to treatment discontinuation (A) and overall survival (B) of patients treated with imatinib in the adjuvant setting using standard censoring. Kaplan-Meier curves of time to treatment discontinuation (C) and overall survival (D) for patients treated with imatinib in the adjuvant setting assuming all patients lost to follow-up (LTFU) were deceased. Dashed line demonstrates median OS. Number at risk at each time point are shown immediately below each curve. Confidence intervals are indicated by shading along the curve.

© 2024 Briercheck EL et al. *JAMA Network Open*.

eFigure 3. Pearson Correlation Test of Country Life Expectancy vs Age at Imatinib Approval

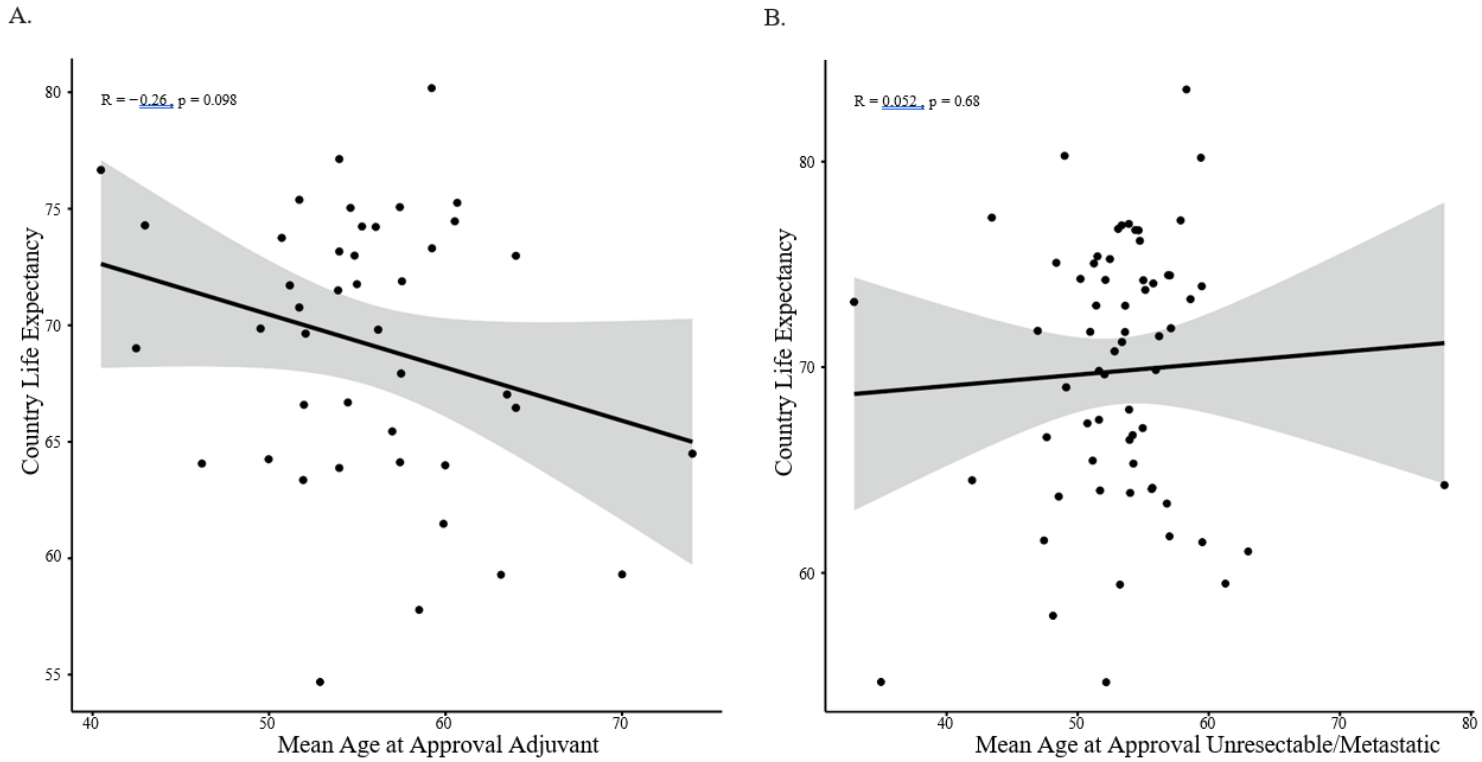

(A) adjuvant and (B) metastatic cohorts.

© 2024 Briercheck EL et al. *JAMA Network Open*.
