# Supplementary material for: Treatment Access for Gastrointestinal Stromal Tumor in Predominantly Low- and Middle-Income Countries
Source: JAMA Netw Open. 2024 Apr 3;7(4):e244898. doi: 10.1001/jamanetworkopen.2024.4898 (PMC10993077; doi:10.1001/jamanetworkopen.2024.4898)
Supplement: Supplement 2. — Data Sharing Statement [file jamanetwopen-e244898-s002.pdf]

# Data Sharing Statement

Briercheck. Treatment Access for Gastrointestinal Stromal Tumor in Predominantly Low- and Middle-Income Countries. *JAMA Netw Open*. Published April 03, 2024.
doi:10.1001/jamanetworkopen.2024.4898

**Data**
**Data available:** Yes
**Data types:** Deidentified participant data
**How to access data:** Deidentified data will be made available upon request by the corresponding author (michael_wagner@dfci.harvard.edu) after approval by The Max Foundation (pato@themaxfoundation.org).
**When available:** With publication

**Supporting Documents**
**Document types:** None

**Additional Information**
**Who can access the data:** Researchers whose proposed use of the data has been approved.
**Types of analyses:** For purposes approved by The Max Foundation.
**Mechanisms of data availability:** after approval of a proposal
